# Entropy Value-Based Pursuit Projection Cluster for the Teaching Quality Evaluation with Interval Number

**DOI:** 10.3390/e21020203

**Published:** 2019-02-21

**Authors:** Ming Zhang, Jinpeng Wang, Runjuan Zhou

**Affiliations:** School of Civil Engineering, Anhui Polytechnic University, Wuhu 241000, China

**Keywords:** teaching quality evaluation, projection pursuit cluster, entropy value, Monte Carlo simulation, interval number

## Abstract

The issue motivating the paper is the quantification of students’ academic performance and learning achievement regarding teaching quality, under interval number condition, in order to establish a novel model for identifying, evaluating, and monitoring the major factors of the overall teaching quality. We propose a projection pursuit cluster evaluation model, with entropy value method on the model weights. The weights of the model can then be obtained under the traditional real number conditions after a simulation process by Monte Carlo for transforming interval number to real number. This approach can not only simplify the evaluation of the interval number indicators but also give the weight of each index objectively. This model is applied to 5 teacher data collected from a China college with 4 primary indicators and 15 secondary sub-indicators. Results from the proposed approach are compared with the ones obtained by two alternative evaluating methods. The analysis carried out has contributed to having a better understanding of the education processes in order to promote performance in teaching.

## 1. Introduction

The evaluation of teaching quality is an important part of teaching management, and the teachers’ teaching quality is directly related to the cultivation of high quality students [1,2,3]. Therefore, the evaluation of teachers’ teaching quality helps teachers to improve their self-improvement, promote the quality of teaching and the level of education, and finally achieve the goal of improving the quality of teaching [4,5]. International colleges and universities attach great importance to the evaluation of teachers. It not only has a very detailed evaluation and evaluation procedure, but also sets up the corresponding evaluation agency to carry out the evaluation work. In this context, a series of qualitative and quantitative evaluation methods are proposed [6,7,8].

Our work is mainly limited to the quantitative evaluation of teaching quality, and the evaluation index data are given by experts in the form of interval numbers. Due to the complexity and fuzziness of objective world, the experts involved in the teaching quality evaluation have difficulty giving accurate evaluation index values, but only the approximate range of the interval form number [9]. The study of interval number and the sorting problem have more important theoretical and practical value, which has gradually aroused the attention of scholars, and some applications have been applied in the teaching quality evaluation [10].

The motivation of this paper lies in the fact that the traditional method calculation process is too complicated in the evaluation of interval number form, the calculation and evaluation process of weight needs to be carried out in stages, and the evaluation result has not been tested for statistical significance. This paper intends to study the following three questions: how to determine the weight of the index objectively, how to easily evaluate the interval number, and how to test the significance of the evaluation results. The literature review is as follows. Liu et al. used interval number to indicate the evaluation value, and established the fuzzy sorting method based on group consensus for teaching quality evaluation in colleges and universities [11]. Zhang described each index interval number according to the characteristics of evaluation on classroom teaching quality, and the ideal point method was adopted to evaluate the quality of classroom teaching. The case study results illustrated the rationality and validity of the proposed method [12]. Zhao investigated the multiple attribute decision making problems with interval-valued intuitionistic fuzzy information, established an optimization model based on the basic ideal of traditional Technique for Order of Preference by Similarity to Ideal Solution (TOPSIS), and illustrated the developed procedures for evaluating the college class teaching quality [13]. Jahanshahloo et al. extended the concept of TOPSIS to develop a methodology for solving multi-attribute decision-making problems with fuzzy data and interval data respectively [14,15]. Fouskakis proposed a Bayesian hierarchical beta regression model with a Dirichlet prior on the model coefficients. The coefficients of the model can then be interpreted as weights and thus they measure the relative importance that students give to the different attributes [16]. Gu et al. analyzed the importance of teaching valuation and the phenomenon of distortion of teaching evaluation data, and proposed a cluster evaluation model for research on teaching evaluation data by using an improved K-Mode clustering method [17].

However, aforementioned studies did not consider the method of evaluating the weight of different indexes, and the weights of each index are equal. For this situation, Dong et al. established multiple objects programming model to obtain each evaluation index weights, and then they gave the priorities of teachers [18]. Chen presented a framework for teaching performance evaluation based on the combination of fuzzy Analytic Hierarchy Process (AHP) and fuzzy comprehensive evaluation method, and the factor and sub-factor weights were estimated by the extent analysis fuzzy AHP method [19]. Shen et al. constructed the Evaluation Index system of Nursing Simulation teaching Quality and determined the weight of each indicator by the combination of Delphi method and AHP method [20].

The weighting process is critical for the results of the teaching quality evaluation and it should be guaranteed that the outcomes of the evaluation are not manipulated by those carrying out the assessment [16]. At the same time, most of the interval number method is complicated and tedious, and sometimes the loss of information in dealing with the problem leads to the distortion of evaluation.

The purpose of this paper is to propose a method framework for simplifying the evaluation of teaching quality expressed in the form of interval numbers, at the same time, improve the objectivity of the weight calculation process, and test the significance of the evaluation results. The main steps of the method are as follows. Firstly, we use the information entropy method [21,22] to calculate the weight of each index, and minimize the effect of subjective factors. Secondly, we use the Monte Carlo method to turn the interval into a common real number. The evaluation index system of real number is evaluated, which can be simplified. Thirdly, the data of the teaching quality index of the teaching quality are evaluated by using the projection pursuit clustering method (PPC) [23,24]. Fourthly, the statistical results of each evaluation object are obtained after multiple simulations of interval number.

The rest of this paper is organized, as follows: Section 2 gives the methodological framework and introduces the main principles of entropy value and PPC; Section 3 gives the results of a case study and a discussion, and the evaluation is made in the case of different random simulations; and, Section 4 summarizes the main research results of this paper, and by calculating the sorting of the quality evaluation of teachers’ teaching, it is beneficial to improve the teaching quality of teachers in the future, and has certain reference value for other similar evaluation problems.

## 2. Materials and Methods

### 2.1. Monte Carlo Simulation for Interval Teaching Quality Index

Let $lb_{hj}$ and $ub_{hj}$ be the lower bound and upper bound of the *j*th index of the *h*th teacher in interval number, and $x_{ij}^{0}$ be the *j*th index of the *i*th random sample, where *i* = 1, 2, …, *k* × *n*; *j* = 1, 2, …, *m*; *h* = 1, 2, …, *k*; *n* is the number of random simulations of each scheme, *m* is the number of index, and *k* is the teacher number.

The random number of the uniform distribution is generated by the use of Equation (1) below. The number of random simulations for each teacher is selected as 5, 10, 50, 100, 500, and 1000, respectively, to make the random sampling results statistically significant.

(1) $$x_{ij}^{0}=lb_{hj}+\left(ub_{hj}-lb_{hj}\right)\times \mathrm{rand}()$$

where rand() is a function that can generate [0,1] uniform random number.

It should be noted that the simulation process in this paper is only to simplify the evaluation index data expressed in the form of interval number, not to obtain the virtual data of a teacher.

### 2.2. Entropy Value of Evaluation Index Weight

According to the maximum entropy principle [21,22], the index weight of the projection vector distribution should have the maximum entropy. The larger the entropy, the less the constraint and the assumption that people add.

(2) $$H_{w}=-\frac{1}{\ln\left(m\right)}\overset{m}{\underset{j=1}{\sum }}w_{j}\ln\left(w_{j}\right)$$

where, *m* is the number of index; *w* = (*w*_1_, *w*_2_, …, *w_m_*) is the weight of each index calculated by the following equation:

(3) $$w_{j}=a_{j}^{2}\;\left(j=1,2,\cdots ,m\right)$$

where, *a* (*a*_1_, *a*_2_, …, *a_m_*) is the *m*-dimensional unit projection vector taken as the optimization variable, which is solved by the optimization method based on the criterion of the maximum density within the class, the distance between classes and the entropy value.

### 2.3. Projection Pursuit Cluster

Let *z* (*z*_1_, *z*_2_, …, *z_m_*) be the projection eigenvalue set which represent the one-dimensional projection eigenvalue of $x_{ij}$, calculated as follows:

(4) $$z_{i}=\overset{m}{\underset{j=1}{\sum }}a_{j}x_{ij}\;\left(i=1,2,\cdots ,k\times n\right)$$

According to the projection vector, the weight of each index can be calculated by the following equation.

The PPC theory requires that the distribution of the projection eigenvalue *z*(*i*) is as dense as possible, and it is best to build up a number of points; and, the overall projection dots are as scattered as possible. Based on this concept, the projection indicator function *Q*(*a*) can be constructed as:

(5) $$Q\left(a\right)=S_{z}\times \;D_{z}\times H_{w}$$

where, *S_z_* is the standard deviation of *z*(*i*) series; *D_z_* is the local density of *z*(*i*); and *H_w_* is the entropy of index weights. Those formulas are shown as:

(6) $$S_{z}=\sqrt{\overset{k\times n}{\underset{i=1}{\sum }}{\left(z\left(i\right)-E_{z}\right)}^{2}/(k\times n-1)}$$

(7) $$D_{z}=\overset{k\times n}{\underset{i=1}{\sum }}\overset{k\times n}{\underset{i=1}{\sum }}\left(R-r_{ij}\right)u\left(R-r_{ij}\right)$$

where, *E_z_* is the mean of *z*(*i*) series; *R* = 0.1*S_z_* denotes the windows radius of local density; *r_ij_* = |*z*(*i*) − *z*(*j*)|; and *u*(*t*) is the unit step function, which is equal to 1 when *t* ≥ 0, and 0 when *t* < 0.

When the value of *Q*(*a*) reaches a maximum, it obtains the optimal projection vector a* and clustering results. Then the model can be described as a nonlinear optimization question whose formula is given in the following equation:

(8) $$\{\begin{array}{c} \max Q\left(a\right)\\ s.t.\;\|a\|=1 \end{array}$$

In order to solve the above optimization problem and obtain the optimal projection vector a*, we use accelerated genetic algorithms to solve it [25,26]. The optimal projection value series $z_{i}^{*}$ is calculated according to Equation (4) by the optimal projection vector a* that is obtained by Equation (8). The mean and standard deviation of each scheme is calculated, and the Duncan significance test is carried out to compare the mean of each scheme in order to guide the practice of policy.

### 2.4. The Index System of Teaching Quality Evaluation

The index system of teaching quality evaluation in a university has 4 indicators and 15 sub- indicators, as shown in Table 1.

Five grades were used to set up the comments for evaluation: {excellent, very good, good, fair, poor} and the corresponding comment to make the index quantitative in interval number form: {[0.9, 1], [0.8, 0.9], [0.7, 0.8], [0.6, 0.7], [0, 0.6]} were also given by Ref. [12].

In order to facilitate the comparison of the results, our case study data are collected from the literature [12] with 5 alternative teachers *T*1, *T*2, *T*3, *T*4, *T*5, as shown in Table 2.

## 3. Results and Discussions

### 3.1. Monte Carlo Simulation of Interval Number

**Figure 1:**
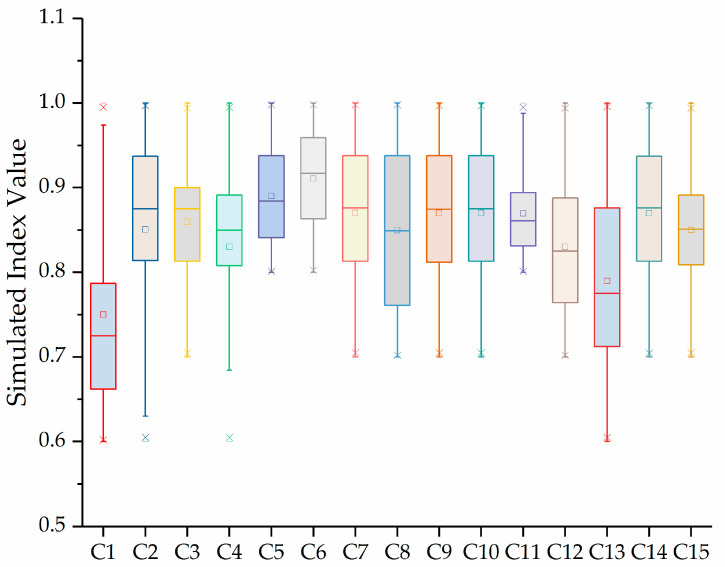
Box-plot of each index value under 1000 simulation times.

According to the Equation (1), the interval number of Table 1 was simulated 5, 10, 50, 100, 500, and 1000 times in sequence, and a real number form evaluation index value were then obtained. The statistical results of each index under 1000 simulation times were shown in Figure 1.

Figure 1 shows that the simulation results of the various sub-indicators of each teacher are between 0.8-0.9 corresponding to the comments “very good”. The indicator values of each teacher are closer, so further evaluation is necessary to determine which teacher is better.

### 3.2. Results of the Indicator Weights of Projection Pursuit Cluster

The random samples were optimized by accelerating genetic algorithm. The projection vector of the 15 indicators was optimized, then each indicator weight was obtained by Equation (3), as shown in Table 3.

Column 3–8 in Table 3 are the weights under each simulation times. As the simulation times increase from 5 to 1000, the weight of each index is gradually stabilized. The weight of each indicator in literature [12] (see Column 2 in Table 3) is determined by minimizing the objective of the weighted distance of the teachers and the ideal teacher. As seen from the column 2, the difference between the weight values of each indicator is small, such as C7, C9, C10, C11, and C14, and they have the same weight 0.0533. From the weights under 1000 simulation times, it shows that sub-indicator C13 is the primary factor to the teaching quality evaluation, while literature [12] is the C1. It is obvious that the effective organization classroom to realize the interaction with the students can reflect the teaching performance.

**Figure 2:**
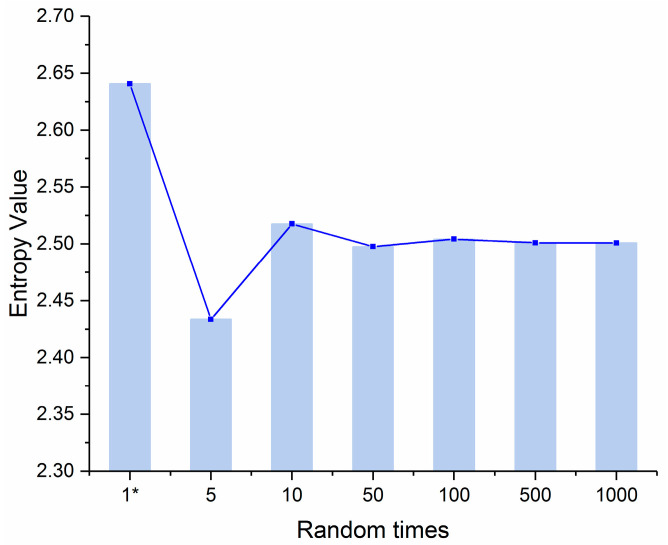
Entropy value of the weight distribution under each random simulation times. (Note: 1* is the entropy value calculated by the index weights from literature [12]).

The information entropy value obtained by the distribution of the index weights is calculated in Figure 2.

Figure 2 shows two results. One is that the information entropy value under each simulation random times increases with the stability of the weight distribution. According to the principle of maximum entropy theory, the probability distribution with the maximum entropy has the minimum uncertainty. Therefore, increasing the simulation times can reduce the uncertainty of the evaluation results. The other result from Figure 2 is that the entropy value from literature [12] has the largest value which seems that the weight uncertainty of literature [12] is the smallest. However, it is actually caused by the shortcomings of the weight determination method in the literature [12]. By observing the data in column 2 of Table 3, it can be seen that the weight of literature [12] has the tendency of equal weight, such as C7, C9, C10, C11, and C14. This shows that the weight calculation method in the literature [12] does not reflect the distribution of evaluation data of each index, and the information entropy calculated according to this weight is not comparable. On the contrary, there is no large amount of the same weight in the various indexes of this paper, which mainly reflects the objective differences of each index.

### 3.3. Evaluation of Each Alternative Teacher

#### 3.3.1. Scatter Plot of Each Teacher

**Figure 3:**
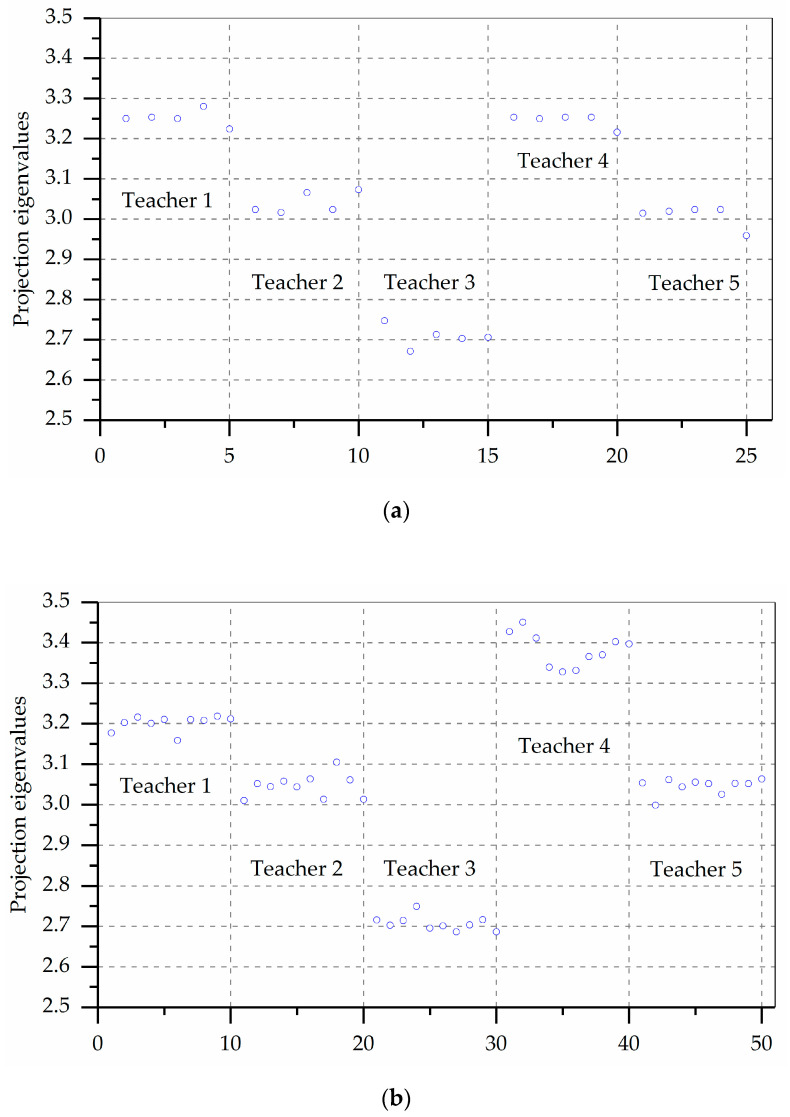

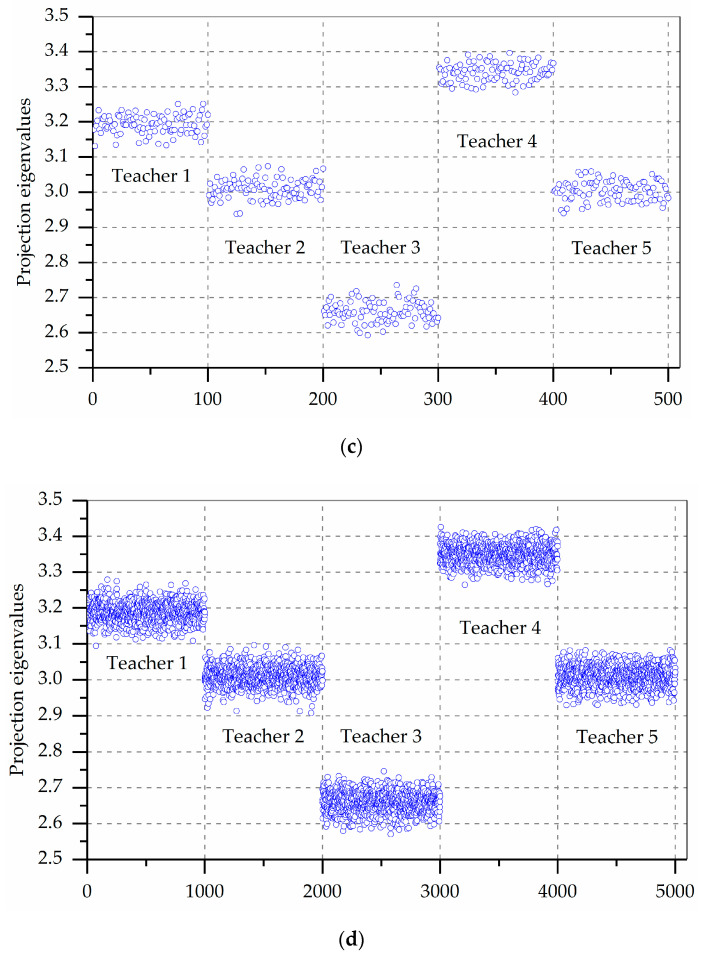
Scatter plot of projection eigenvalues of random samples for each scheme. (**a**) 5 simulation times results; (**b**) 10 simulation times results; (**c**) 100 simulation times results; and, (**d**) 1000 simulation times results.

The projected eigenvalues of each teacher are calculated by using Equation (3), and the projected eigenvalue scatter diagram is plotted (Figure 3) under each simulation times which can show the characteristics of the clustering and dispersion of each projection eigenvalue. Due to space limitations, Figure 3 only shows 5, 10, 100, and 1000 simulation times. Each blue circle represents the value of a simulation. The larger the circle’s projection value is, the higher the corresponding teacher evaluation grade is.

Figure 3 shows that all alternative teachers display clear aggregation characteristics when the simulation times increases. Overall, the projection eigenvalues of the *Teacher 4* are the largest, followed by *Teacher 1*, *Teacher 2*, *Teacher 5*, and *Teacher 3*. If the evaluation solution is made using a small number of decision data, the optimal evaluation results are likely to occur in *T*1 > *T*4 > *T*2 > *T*5 > *T*3 which cause the evaluation results to be more inaccurate, so it is necessary to simulate the calculation multiple times.

However, Figure 3 is only a qualitative comparison of the pros and cons of all alternative teachers. We additionally need to make quantitative comparisons of each teacher.

#### 3.3.2. Statistical Analysis of Projection Eigenvalues

The projection eigenvalues of each teacher are analyzed and the Duncan test method is used to make a significance test, as shown in Table 4.

Table 4 shows that the mean and the standard deviation of random evaluation samples are stable with the increase of simulation times. At the same time, the less the simulation times, the more the ranking of evaluation results will change. When simulation times are 5, the statistical results showed a large difference compared to the results when 1000 simulations are performed. This indicates that the uncertainty of the evaluation is very large and leads to inaccurate decision results, when using a small number of data or directly using the original interval to make an evaluation. Therefore, we suggest evaluating by simulating the indicator values of all alternative teachers, thereby reducing the uncertainty of the evaluation results.

According to the results of the Duncan test, the significance results showed that *T4* is better than *T1*, that *T1* is better than *T2* and *T5* who have the same performance, and that *T2* and *T5* are better than *T3* under the significance level of α = 0.01. In conclusion, the significance test results show that the optimal teacher is *Teacher 4*.

### 3.4. Discussion of the Evaluation Results

#### 3.4.1. Comparisons with Other Methods

In this section, we conclude our experimental results with a comparison of the proposed method with TOPSIS model as presented in Zhang [12]. Results under the two methods can be directly compared, since, as we applied the same data. Even though the two models have a different structure, results are similar, namely *T*4 > *T*1 > *T*2 = *T*5 > *T*3; the larger differences that are noticed are in the weight of indicators (see Table 3) and the uncertainty of the evaluation results. The teacher’s rank results drawn under the two approaches are similar.

#### 3.4.2. Advantages of the Proposed Method

(1) Simple calculation process. By using Monte Carlo stochastic simulation, each teacher’s evaluation index in interval number form is converted into an ordinary real number, which can effectively reduce the complexity of the evaluation calculation. The evaluation process can be evaluated directly by the conventional evaluation method, and it is not necessary to transform it into the evaluation model of interval number.

(2) The weight calculation can be synchronized with the evaluation calculation. The PPC model compresses the high dimensional data to low dimensional, and the weight of each indicator can be obtained by optimizing the object function. This weight is derived from the distribution of the evaluation index, so it is an objective weight. Meanwhile, entropy value of the indicator weight can further reduce the uncertainty of the subjective hypothesis in the calculation process of the weight.

(3) The comparison results of the evaluation of each teacher are statistical. Because the evaluation value of each teacher is the interval value form data given by the evaluation expert, the volatility and uncertainty of the evaluation results will be large, so it is necessary to introduce the statistical analysis. Table 4 shows that the simulation calculation of small amounts of data is different from that of large numbers of data, and there is a greater uncertainty. This paper adopts the simulation idea proposed in this paper, which can be used to measure the significant difference between the evaluation results.

## 4. Conclusions

In this paper, a projection pursuit cluster method based on entropy value is used to evaluate and monitor the major indicators of the overall teaching quality. The proposed method uses the Monte Carlo simulation to convert the interval number to the real number of each indicator value, and the weight of each indicator can also be obtained by the PPC model.

Regarding the results from literature [12] and each simulation time, we observe some differences in the optimal teacher. The different evaluation results show that the uncertainty is a vital factor that must be considered in the evaluation process. According to the 1000 simulation evaluation results, we obtain the optimal *Teacher* 4, *T*4 > *T*1 > *T*2 = *T*5 > *T*3. The innovation lies in the fact that the proposed method simplifies the multi-index evaluation calculation in the form of interval number, makes the weight calculation result more reliable, and tests the significance of the evaluation result. It also provides user-friendly inferences, direct significance interpretations, natural ways for the implementation of the interval number, capability for a continuous assessment, and monitoring. In future research, we intend to use the proposed evaluation method to solve the problems of other uncertainty teaching quality evaluation, such as triangle fuzzy number, gray number, and so on.

## Figures and Tables

**Table 1:** Meanings of each indicator of teaching quality evaluation [12].

Indicators	Sub-Indicators
Teaching requirement	Following the syllabus and teaching plan strictly (C1)
	Well-dressed, dignified, punctual (C2)
	Demonstrating enthusiasm and supports (C3)
	Clear, logical, and innovative documentation (C4)
Teaching content	Clear goals and objectives (C5)
	Accurate concepts, full content, and proper difficulty (C6)
	Analyzing the latest research results (C7)
	Focus on the connection between theory and practice (C8)
Teaching method	Focus on the way of thinking and ability of students (C9)
	Encourage students to express their original views (C10)
	Providing appropriate feedback and teaching by aptitude (C11)
	Using a variety of appropriate media/approaches to present content (C12)
	Effectively handle the teaching process (C13)
Classroom condition	Students are serious and focused (C14)
	Excellent classroom teaching discipline (C15)

**Table 2:** Five teacher’s index value.

Sub-Indicators	*T*1	*T*2	*T*3	*T*4	*T*5
C1	[0.7, 0.8]	[0.7, 0.8]	[0.6, 0.7]	[0.9, 1.0]	[0.6, 0.7]
C2	[0.8, 0.9]	[0.9, 1.0]	[0.6, 0.7]	[0.9, 1.0]	[0.8, 0.9]
C3	[0.9, 1.0]	[0.8, 0.9]	[0.7, 0.8]	[0.9, 0.9]	[0.8, 0.9]
C4	[0.9, 1.0]	[0.8, 0.9]	[0.6, 0.7]	[0.8, 0.9]	[0.8, 0.9]
C5	[0.9, 1.0]	[0.8, 0.9]	[0.8, 0.9]	[0.9, 1.0]	[0.8, 0.9]
C6	[0.9, 1.0]	[0.8, 0.9]	[0.8, 0.9]	[0.9, 1.0]	[0.9, 1.0]
C7	[0.8, 0.9]	[0.7, 0.8]	[0.8, 0.9]	[0.9, 1.0]	[0.9, 1.0]
C8	[0.9, 1.0]	[0.9, 1.0]	[0.8, 0.9]	[0.7, 0.8]	[0.7, 0.8]
C9	[0.8, 0.9]	[0.7, 0.8]	[0.8, 0.9]	[0.9, 1.0]	[0.9, 1.0]
C10	[0.9, 1.0]	[0.8, 0.9]	[0.7, 0.8]	[0.9, 1.0]	[0.8, 0.9]
C11	[0.8, 0.9]	[0.8, 0.9]	[0.8, 0.9]	[0.8, 0.9]	[0.9, 1.0]
C12	[0.9, 1.0]	[0.8, 0.9]	[0.7, 0.8]	[0.8, 0.9]	[0.7, 0.8]
C13	[0.8, 0.9]	[0.7, 0.8]	[0.6, 0.7]	[0.9, 1.0]	[0.7, 0.8]
C14	[0.8, 0.9]	[0.9, 1.0]	[0.7, 0.8]	[0.9, 1.0]	[0.8, 0.9]
C15	[0.8, 0.9]	[0.8, 0.9]	[0.7, 0.8]	[0.9, 1.0]	[0.8, 0.9]

**Table 3:** Weights of each index under each simulation random times.

Sub-Indicators	Ref. [12]	Simulation Times					
		5	10	50	100	500	1000
C1	0.1333	0.0169	0.1478	0.1041	0.1206	0.1064	0.1066
C2	0.0667	0.0354	0.1248	0.1093	0.0961	0.1141	0.1142
C3	0.0667	0.0631	0.0485	0.0790	0.0801	0.0807	0.0806
C4	0.0800	0.1386	0.0765	0.1034	0.1160	0.1020	0.1021
C5	0.0400	0.0552	0.0698	0.0395	0.0429	0.0429	0.0427
C6	0.0267	0.0520	0.0618	0.0444	0.0582	0.0475	0.0474
C7	0.0533	0.0243	0.0842	0.0437	0.0421	0.0436	0.0437
C8	0.0667	0.0476	0.0005	0.0002	0.0003	0.0003	0.0003
C9	0.0533	0.0620	0.0129	0.0341	0.0408	0.0474	0.0474
C10	0.0533	0.2121	0.0818	0.0918	0.0817	0.0908	0.0909
C11	0.0533	0.0012	0.0167	0.0064	0.0035	0.0043	0.0043
C12	0.0800	0.1230	0.0551	0.0462	0.0555	0.0419	0.0419
C13	0.1067	0.0665	0.0610	0.1464	0.1355	0.1435	0.1434
C14	0.0533	0.0602	0.0779	0.0698	0.0577	0.0630	0.0629
C15	0.0667	0.0419	0.0807	0.0819	0.0688	0.0716	0.0717

**Table 4:** Results of statistical characters and significance analysis.

Times	*T*1	*T*2	*T*3	*T*4	*T*5
5	3.252 ± 0.020 aA	3.040 ± 0.027 bB	2.708 ± 0.027 dC	3.245 ± 0.016 aA	3.008 ± 0.027 cB
10	3.201 ± 0.019 B	3.046 ± 0.029 C	2.707 ± 0.018 D	3.382 ± 0.042 A	3.046 ± 0.020 C
50	3.181 ± 0.032 B	3.014 ± 0.027 C	2.655 ± 0.033 D	3.345 ± 0.023 A	3.011 ± 0.028 C
100	3.193 ± 0.027 B	3.01 ± 0.027 C	2.658 ± 0.029 D	3.342 ± 0.025 A	3.004 ± 0.027 C
500	3.187 ± 0.028 B	3.013 ± 0.029 C	2.662 ± 0.030 E	3.345 ± 0.026 A	3.007 ± 0.029 D
1000	3.188 ± 0.028 B	3.009 ± 0.028 C	2.659 ± 0.029 D	3.349 ± 0.028 A	3.009 ± 0.029C

Note: abcd is the significance level under α = 0.05, and ABCD under α = 0.01.
